# Training cessation and subsequent retraining of a world‐class female Olympic sailor after Tokyo 2020: A case study

**DOI:** 10.14814/phy2.15593

**Published:** 2023-02-07

**Authors:** Yuko Ishida, Takaki Yamagishi, Iñigo Mujika, Mariko Nakamura, Eiko Suzuki, Daichi Yamashita

**Affiliations:** ^1^ Sports Medical Center Japan Institute of Sports Sciences, Japan High Performance Sport Center Tokyo Japan; ^2^ Department of Sport Science and Research Japan Institute of Sports Sciences, Japan High Performance Sport Center Tokyo Japan; ^3^ Department of Physiology, Faculty of Medicine and Nursing University of the Basque Country Leioa Spain; ^4^ Exercise Science Laboratory, School of Kinesiology, Faculty of Medicine Universidad Finis Terrae Santiago Chile

**Keywords:** performance decline, sailing, training modality, transition phase

## Abstract

Olympic sailing is a complex sport where sailors are required to predict and interpret weather conditions while facing high physical and physiological demands. While it is essential for sailors to develop physical and physiological capabilities toward major competition, monitoring training status following the competition is equally important to minimize the magnitude of detraining and facilitate retraining. Despite its long history in the modern Olympics, reports on world‐class sailors' training status and performance characteristics across different periodization phases are currently lacking. This case study aimed to determine the influence of training cessation and subsequent retraining on performance parameters in a world‐class female sailor. A 31‐year old female sailor, seventh in the Women's Sailing 470 medal race in Tokyo 2020, completely stopped training for 4 weeks following the Olympics, and resumed low‐intensity training for 3 weeks. Over these 7 weeks, 12.7 and 5.3% reductions were observed in 6 s peak cycling power output and jump height, respectively, with a 4.7% decrease in maximal aerobic power output. Seven weeks of training cessation‐retraining period induced clear reductions in explosive power production capacities but less prominent decreases in aerobic capacity. The current findings are likely attributed to the sailor's training characteristics during the retraining period.

## Graphical abstract

In this study, we investigated the impact of training cessation and subsequent retraining on physiological performance in a world‐class female Olympic sailor after the 2020 Tokyo Olympics. We observed clear reductions in explosive power production capacities (i.e., peak power output achieved in a 6 s cycle test and jump height) over 7 weeks of training cessation‐retraining period, whereas decreases in aerobic capacities were less prominent. The current findings are likely attributed to the sailor's training characteristics during the retraining period where she mainly performed low‐intensity aerobic cycling.

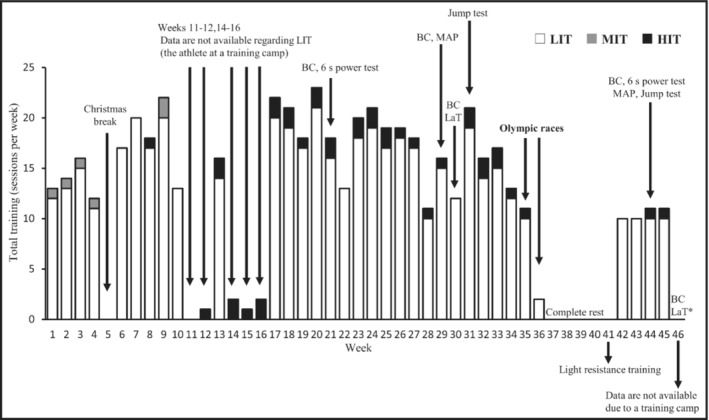

## 1 INTRODUCTION

Olympic sailing is a complex sport where sailors are required to predict and interpret weather conditions while facing high physical and physiological demands (Bojsen‐Møller et al., 2015). Physical requirements of Olympic sailing have been extensively investigated (Bojsen‐Møller et al., 2015) and well‐developed aerobic and anaerobic capacities of elite sailors (Bojsen‐Møller et al., 2007; Vangelakoudi et al., 2007) suggest the importance of training those capacities in this sport. While optimal preparation is essential for winning a major championship (Mujika, 2014), physical and mental recovery during a transition period seem to be equally important in elite sports. However, the duration of training cessation or the magnitude of detraining during a transition phase have been shown to negatively impact subsequent performance in elite/sub‐elite athletes (Godfrey et al., 2005; Mujika et al., 1995). For example, 8 weeks of complete inactivity following the Olympic games induced clear reductions in key endurance parameters (e.g., peak oxygen uptake, power outputs at blood lactate concentrations of 2 and 4 mmol/L) in an Olympic champion rower, which required prolonged (20 weeks) retraining to regain those adaptations (Godfrey et al., 2005). Likewise, the magnitude of detraining following an off‐season has been negatively associated with performance progression in the subsequent season in highly trained swimmers (Mujika et al., 1995). Moreover, decreased aerobic (García‐Pallarés et al., 2009) and neuromuscular (García‐Pallarés et al., 2010) functions were reported in world‐class kayakers following a 5‐week transition period, with greater declines observed in complete training cessation compared to reduced training. Furthermore, divergent decay profiles of neuromuscular functions following resistance training cessation have been documented in elite/sub‐elite cyclists with more rapid decreases in explosive power production capacities compared to muscular performance at lower contraction velocities (Bláfoss et al., 2022; Rønnestad et al., 2016). These findings highlight the importance of the transition period, and the time course of performance declines can be parameter‐dependent in the aforementioned sports. Nevertheless, no comparable data are available in Olympic sailing despite its long history in the modern Olympics. Moreover, it would be important to have a deeper understanding of how a world‐class athlete responds to training cessation and subsequent retraining, as available evidence on such a topic is extremely limited (Godfrey et al., 2005). Therefore, this study aimed to examine the influence of training cessation and subsequent retraining on multiple performance parameters in a world‐class female sailor.

## 2 METHODS

### 2.1 Participant

A 31‐year old female Olympic sailor (470 crew) who placed seventh in the Women's Sailing 470 medal race in Tokyo 2020 participated in this study. She and her partner (helmsman) had previously placed fifth at the 2016 Rio de Janeiro Olympics, and had won gold and silver medals at the Sailing World Championships Aarhus 2018 and 470 World Championship Enoshima 2019, respectively. The participant can thus be classified as a world‐class athlete (McKay et al., 2022). This study was approved by the Institutional Research Ethics Committee (2021–064) and a signed informed consent was obtained from the athlete.

### 2.2 Overview of the study

**FIGURE 1:**
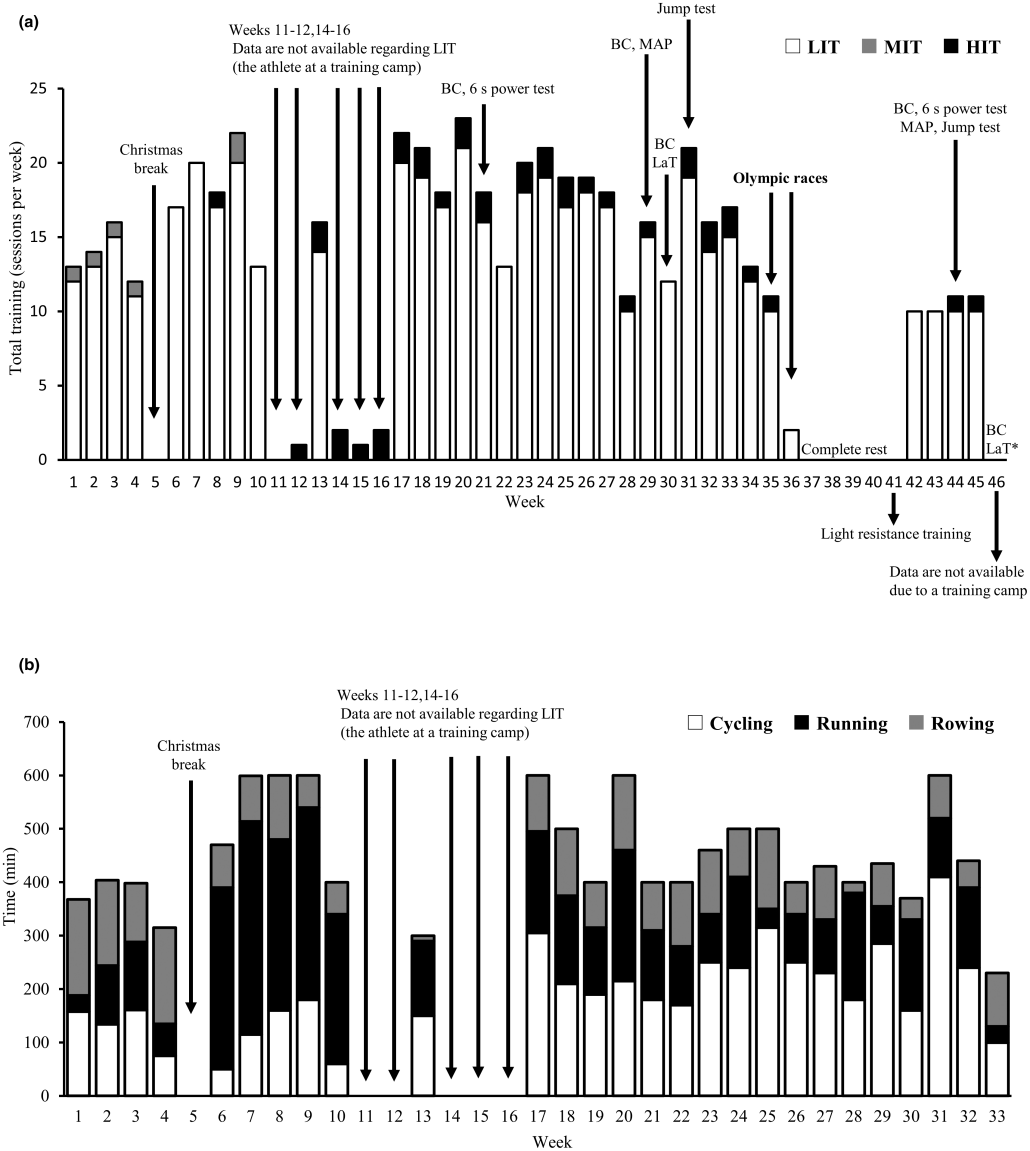
Weekly training frequency performed by the athlete over 46 weeks including preparatory, competitive, transition, and retraining periods (a). Weekly training volume and the distribution of exercise modalities of low‐intensity training prior to the 2020 Tokyo Olympics (b). Note that on‐water sailing training sessions were not included in the graphs. BC, body composition assessment; HIT, high‐intensity training; LaT, blood lactate test; LIT, low‐intensity training; MAP, maximal aerobic power output; MIT, moderate‐intensity training. *The post‐Olympic LaT was conducted immediately after the sailor had returned from the training camp.

This case study covers the data on the sailor's training and performance measurements for 46 weeks. Her weekly training frequency and the distribution of intensity (low‐intensity [LIT], moderate‐intensity [MIT], and high‐intensity [HIT] training) are shown in Figure 1a, while weekly training volume (duration) and the distribution of exercise modalities of LIT (cycling, running, and rowing) are provided in Figure 1b. Moreover, the characteristics of cycle MIT and HIT sessions are shown in Table 1. In addition to these training sessions, she also performed on‐water sailing training for 3–5 h per day, 5 days per week on average, depending on weather conditions prior to the Olympics. Her final event at the Olympics was the Women's Sailing 470 medal race held on the 4th of August 2021. Following her final race, she took 4 weeks of complete rest and then resumed her training from September 6th, starting with bodyweight squats and light‐weight bench presses. From the following week, she started low‐intensity aerobic exercise, cycling approximately 300 min per week. She then restarted on‐water sailing training together with the resistance and aerobic training from September 27th. The athlete's body composition, aerobic and anaerobic performances were determined before and after the 2020 Tokyo Olympics (spanning between 15 weeks before and 10 weeks after the Olympics, Figure 1a).

**TABLE 1:** Training variables during cycle MIT and HIT sessions.

Parameters	MIT	HIT
Session‐averaged power output (W)	149 ± 5	457 ± 57
Relative intensity (% MAP)	57.6 ± 2.1	177.3 ± 22.0
Session duration (min)	35 ± 0	13 ± 5
Session‐averaged heart rate (bpm)	163 ± 6	164 ± 6

*Note*: MIT consisted of five repetitions of 3 min cycling at 45%–50% of MAP interspersed with 4 min cycling at ≥65% of MAP (i.e., 35 min in total). HIT consisted of 10 repetitions of 10–15 s of high‐intensity cycling interspersed with 30–50 s of passive rest. The athlete performed 21 cycle HIT sessions in total: 2 sets of 10 repetitions separated by 5 min of rest (i.e., 20 repetitions in total) during the first 11 sessions, whereas only 10 repetitions were completed during the remaining 10 sessions.

Abbreviations: HIT, high‐intensity training; MAP, maximal aerobic power output; MIT, moderate‐intensity training.

### 2.3 Body mass and composition

Body mass and composition were determined before (April 20th, June 15th and 22nd) and after (September 27th and October 14th) the Olympics on a bio‐impedance meter (Inbody770, Inbody Japan, Tokyo, Japan), where body mass and fat percentage were recorded to the nearest 0.1 kg and 0.1%, respectively. All measurements were completed immediately before performance testing. Her height was measured on five different occasions using a stadiometer (AD‐6228A, A&D Company, Limited, Tokyo, Japan) prior to the current study: 177.1 ± 0.3 cm (mean ± standard deviation).

### 2.4 Maximal incremental cycle test

The athlete performed maximal incremental cycle tests to determine maximal aerobic power output (MAP) on a cycle ergometer (Wattbike pro, Wattbike Ltd, Nottingham, UK) before (June 15th), and after (September 27th) the Olympics. The test commenced at an initial power output of 60 W, with subsequent 20 W increments every minute until volitional exhaustion or until the athlete could no longer maintain the required work rate despite strong verbal encouragement (Zhang et al., 1991). The athlete was allowed to stand on the pedals if she felt difficulty in producing required power with a seated position. Heart rate was recorded throughout using a heart rate sensor (Polar H10, Polar Electro, Kempele, Finland), and maximal heart rate (HRmax) was defined as the highest value recorded over a 1‐s period (Bentley & McNaughton, 2003). MAP was defined as the average power output over the final 60 s of the test (Bentley & McNaughton, 2003).

### 2.5 Submaximal cycle test

Before (June 22nd) and after (October 14th) the Olympics, the athlete also performed submaximal cycle tests to determine power outputs corresponding to blood lactate concentrations of 2 and 4 mmol/L on a cycle ergometer (Powermax VIII, Konami Holdings Corporation, Tokyo Japan). The test commenced at 60 W with 40 W increments every 4 min (Hauser et al., 2014). Blood lactate concentration was determined via earlobe blood samples (Lactate Pro2, Arkray Inc., Kyoto, Japan) during the last 30 s of each stage until the value reached ≥4 mmol/L.

### 2.6 6 s cycle test

The athlete performed all‐out 6 s maximal sprints on a cycle ergometer (Wattbike pro) to determine anaerobic power before (April 20th) and after (September 27th) the Olympics. She first completed a self‐paced warm‐up for at least 5 min, and then cycled maximally for 6 s. The air and magnetic resistances were set to level 3 and level 1, respectively, according to body mass and sex of the athlete. The peak power output achieved over 6 s was defined as anaerobic power (Herbert et al., 2015).

### 2.7 Jump performance test

The athlete conducted jump performance tests before (June 29th) and after (October 1st) the Olympics. On each occasion, she performed three maximal counter‐movement jumps with her arms crossed on her chest with a 30 s rest in between. She was instructed to stand upright and motionless for 1 s then began the movement of the jump on two force platforms (0.9 m × 0.6 m, type 9281; Kistler, Winterthur, Switzerland). Jump height was calculated as previously described (Yamashita et al., 2020) and the highest value was retained for the analysis.

## 3 RESULTS

### 3.1 Body mass and composition

The body mass and percentage body fat values on April 20th, June 15th, June 22nd, September 27th, and October 14th were 70.9 kg and 22.4%, 71.1 kg and 22.1%, 71.5 kg and 22.1%, 70.9 kg and 23.9%, and 70.6 kg and 23.5%, respectively.

### 3.2 Maximal incremental cycle test

Absolute and relative MAP before and after the Olympics were 264 W (3.72 W/kg) and 251 W (3.54 W/kg), respectively (Table 2). The values of HRmax recorded during the incremental test were 189 and 190 beats/min before and after the Olympics, respectively.

**TABLE 2:** Performance parameters before and after the Tokyo 2020 Olympic games.

Parameters	Pre‐Olympics	Post‐Olympics	%change	Cessation‐retraining period before the post‐Olympic tests (wks)
6 s peak power output (W)	943	823	−12.7	7
6 s peak power output (W/kg)	13.30	11.61	−12.7	
MAP (W)	264	251	−4.9	7
MAP (W/kg)	3.72	3.54	−4.7	
Power output at 4 mmol/L (W)	194	189	−2.6	9
Power output at 4 mmol/L (W/kg)	2.72	2.68	−1.4	
Power output at 2 mmol/L (W)	152	154	1.3	9
Power output at 2 mmol/L (W/kg)	2.13	2.18	2.5	
Jump height (cm)	24.5	23.2	−5.3	7

Abbreviation: MAP, maximal aerobic power output.

### 3.3 Submaximal cycle test

Power outputs corresponding to blood lactate concentrations of 2 and 4 mmol/L before the Olympics were 152 W (2.13 W/kg) and 194 W (2.72 W/kg), respectively (Table 2). Corresponding values after the Olympics were 154 W (2.18 W/kg) and 189 W (2.68 W/kg), respectively (Table 2).

### 3.1 6 s cycle test

Anaerobic power achieved before the Olympics was 943 W (13.30 W/kg), versus 828 W (11.61 W/kg) after the Olympics (Table 2).

### 3.2 Jump performance test

Jump height before the Olympics was 24.5 cm versus 23.2 cm after the Olympics (Table 2).

## 4 DISCUSSION

This study examined the impact of training cessation and subsequent retraining on performance parameters in a world‐class sailor, and observed 12.7 and 5.3% reductions in anaerobic power and jump height following the 7‐week training cessation‐retraining period, with slightly reduced aerobic performance (Table 2), and fairly constant body mass and composition over the study period. Previously, 8 weeks of training cessation were reported to decrease power output at peak oxygen uptake by 20%, and those at blood lactate concentrations of 4 and 2 mmol/L by 22 and 27%, respectively in a male Olympic champion rower. Following 8 weeks of retraining, the corresponding power outputs were still 8, 7 and 6% lower than before the Olympics (Godfrey et al., 2005). In the current study, a smaller reduction was observed in MAP after the 7‐week training cessation‐retraining period (Table 2). Moreover, with two additional weeks of retraining, power outputs at 4 and 2 mmol/L were within 3% of the pre‐Olympic values (Table 2). The discrepancy in the magnitudes of reductions between the current and previous (Godfrey et al., 2005) studies is likely explained by the different training cessation periods (i.e., 4 vs. 8 weeks). Nevertheless, the sailor showed greater reductions in explosive power production capacities (i.e., 6 s peak power and jump height) (Table 2), which is somewhat in line with previous studies (Bláfoss et al., 2022; Rønnestad et al., 2016). For instance, Rønnestad et al. (2016) observed greater declines in squat jump (SJ) performance and mean power during a 30‐s Wingate test compared to maximal voluntary contraction (MVC) following 8 weeks of resistance training cessation in elite cyclists. Likewise, Bláfoss et al. (2022) showed that leg extension power and rate of force development (RFD) declined at a faster rate than MVC and 5‐min mean cycling power following 6 weeks of resistance training cessation in sub‐elite cyclists. These findings indicate that training cessation may inhibit muscle performance at higher contraction velocities (e.g., SJ, RFD) more rapidly and severely than it does lower‐velocity performance (e.g., MVC). In the current study, the 4‐week training cessation followed by 3 weeks of low‐intensity retraining caused approximately 13 and 5% declines in 6 s cycle peak power and jump height, respectively (Table 2). Moreover, the complete cessation of on‐water sailing training, which involves very dynamic actions on the dinghy (Bojsen‐Møller et al., 2015), over the same period may also explain some of the declines in the explosive performance. Contrary to the current study, the inclusion of 3 × 30 s sprints in one weekly LIT sessions during a transition period in elite cyclists induced greater repeated 30 s sprint performance compared with a control group who only performed LIT (Almquist et al., 2020). Furthermore, Rønnestad et al. (2010) demonstrated that a single weekly resistance training was effective in maintaining previously‐increased Wingate peak power during 13 weeks of competition period in well‐trained cyclists. Taken together, the inclusion of a small dose of sprint‐type and/or resistance training could have alleviated the decline in anaerobic performance observed in the current study.

## 5 LIMITATIONS

A major limitation of this study is that the time spans between pre‐ and post‐Olympic tests were different among the measured parameters (Figure 1a, Table 2), which makes it difficult to claim that we truly measured the effects of training cessation and subsequent retraining on performance. Nevertheless, the changes of relative 6 s peak power and MAP of the athlete had been 3.0 and 0.7% over several months, respectively, before the pre‐Olympic measurements (when she followed a regular training regimen). Therefore, greater changes in those values and intensity‐dependent decline in cycling performance (i.e., 6 s power > MAP > submaximal power, Table 2) following the Olympics are likely explained, at least partially, by the training cessation‐retraining period.

## 6 PERSPECTIVES

While an athlete needs physical and mental recovery following a major competition such as the Olympic Games, limiting the decline in physiological and performance adaptations could be advantageous for the next competitive season (Mujika et al., 1995). This study indicates that although LIT alone somewhat contributed to maintaining aerobic adaptations, the complete cessation of HIT for 7 weeks diminished anaerobic conditioning in a world‐class sailor. A minimum dose of HIT (e.g., once per week) may alleviate the reduction in anaerobic performance without compromising mental recovery (Almquist et al., 2020; Rønnestad et al., 2010).

## AUTHOR CONTRIBUTIONS

IM, TY, YI, DY, and MN designed the study. YI, ES, and TY planned training programmes for the athlete. DY and MN supervised the progress of the athlete's training. YI and ES collected the data. YI, ES, and TY analyzed the data. IM, TY, and DY interpreted the results. TY wrote the manuscript and all authors critically reviewed it. All authors read and approved the final version of the manuscript.

## CONFLICT OF INTEREST STATEMENT

The authors declare that they have no conflict of interest.

## ETHICS STATEMENT

This study was approved by the Institutional Research Ethics Committee (approval number: 2021–064).
